# Functional regionalization of the differentiating cerebellar Purkinje cell population occurs in an activity-dependent manner

**DOI:** 10.3389/fnmol.2023.1166900

**Published:** 2023-04-27

**Authors:** Alessandro Dorigo, Komali Valishetti, Florian Hetsch, Hideaki Matsui, Jochen C. Meier, Kazuhiko Namikawa, Reinhard W. Köster

**Affiliations:** ^1^Cellular and Molecular Neurobiology, Technische Universität Braunschweig, Braunschweig, Germany; ^2^Cell Physiology, Zoological Institute, Technische Universität Braunschweig, Braunschweig, Germany; ^3^Institute of Pathophysiology, University Medical Center, Johannes Gutenberg University, Mainz, Germany; ^4^Department of Neuroscience of Disease, Brain Research Institute, Niigata University, Niigata, Japan

**Keywords:** zebrafish, cerebellum, Purkinje cell, functional regionalization, calcium imaging, optogenetics, spine density

## Abstract

**Introduction:**

The cerebellum is organized into functional regions each dedicated to process different motor or sensory inputs for controlling different locomotor behaviors. This functional regionalization is prominent in the evolutionary conserved single-cell layered Purkinje cell (PC) population. Fragmented gene expression domains suggest a genetic organization of PC layer regionalization during cerebellum development. However, the establishment of such functionally specific domains during PC differentiation remained elusive.

**Methods and results:**

We show the progressive emergence of functional regionalization of PCs from broad responses to spatially restricted regions in zebrafish by means of in vivo Ca2+-imaging during stereotypic locomotive behavior. Moreover, we reveal that formation of new dendritic spines during cerebellar development using in vivo imaging parallels the time course of functional domain development. Pharmacological as well as cell-type specific optogenetic inhibition of PC neuronal activity results in reduced PC dendritic spine density and an altered stagnant pattern of functional domain formation in the PC layer.

**Discussion:**

Hence, our study suggests that functional regionalization of the PC layer is driven by physiological activity of maturing PCs themselves.

## Significance statement

Purkinje cells, (PC), the sole output neurons of the vertebrate cerebellum are organized into functional regions mediating different behaviors. By stimulating reflexive stereotypic behavior and high-resolution imaging of individual PCs during embryogenesis, we reveal that functional regionalization in the PC layer occurs concomitant with dendritic spine formation. Pharmacological as well as PC autonomous long-term optogenetic silencing of PC activity impairs dendritic spine density as well as functional regionalization of the PC layer. This demonstrates that functional regionalization and thus fine-tuning of PC connectivity is driven by neuronal activity of PCs themselves.

## Introduction

The vertebrate cerebellum is dedicated to control body posture, balance, locomotor control and motor learning among other less well-clarified functions such as processing of sensory and emotional information and predicting dynamic events requiring motor activity (Timmann et al., 2010; Brooks et al., 2015; Khilkevich et al., 2018). Also, cerebellar development and its anatomy is highly conserved among vertebrates, which has been shown in particular for zebrafish and mammals (Wullimann et al., 2011; Hibi and Shimizu, 2012; Matsui et al., 2014). In contrast, the maturation of cerebellar neurons and the refinement of their physiological organization is understudied.

Purkinje cells (PCs) are arranged in a single cell layer and they represent the sole output neurons of the cerebellar cortex. In mammals, PCs are organized into functional domains dedicated to process and control different sensory and locomotive tasks. These functional domains can be clearly outlined and represented in form of a sensory and motor somatotopic map–albeit this map is fractured with topographically discontinuous patches of PCs representing non-adjacent areas of the body (Gibson et al., 1987; Gonzalez et al., 1993; Provini et al., 1998; Schlerf et al., 2010). In zebrafish, a similar regionalization of the PC layer has been shown by efferent connectivity mapping, Ca^2+^-imaging during reflexive behavior and regionalized optogenetic interference with PC physiology (Matsui et al., 2014; Harmon et al., 2017; Knogler et al., 2019). Regionalization in the PC layer appears to be organized predominantly along the rostrocaudal axis with symmetric clusters in the caudal area being responsible for eye and body coordination corresponding to the vermis in the human cerebellum, while PCs in the rostro- and rostra-medial area are dedicated to swimming and turning corresponding to the human cerebellar paravermis (Matsui et al., 2014; Knogler et al., 2019).

Regionalized expression of signal transduction factors during PC differentiation in spatial correspondence to afferent projections and efferent circuitry appear to assemble PCs into coherent groups of roughly defined zones in mammals (Gravel and Hawkes, 1990; Rogers et al., 1999; Sillitoe et al., 2010; Ebner et al., 2012; Vibulyaseck et al., 2015; Takeuchi et al., 2017). In addition, elegant studies in mice have implicated electrophysiological activity of PCs to organize them into PC functional regions with zonal circuitry (White et al., 2014). Yet, until now, a zonal expression of genes in the zebrafish PC population either regionalized or fragmented has not been identified so far.

While, functional regionalization of the PC layer is evident in vertebrates, the developmental appearance of regionalized functional domains is far less understood. The small and nearly transparent zebrafish larvae with the unfolded PC layer are ideal for non-invasive *in vivo* imaging approaches (Weber and Köster, 2013). Hence, we combined triggering of stereotypic swimming movements with measurement of Ca^2+^ transients on the population level in zebrafish larvae on consecutive days to examine the development of functional domains. We also revealed that the formation of dendritic spines correlates with the temporal period of functional regionalization. Moreover, acute inhibition of PC activity interfered with dendritic spine formation and altered functional regionalization of the PC layer. Therefore, activity of PCs seems to be required for their own maturation and functional fine tuning within the PC layer.

## Materials and methods

### Animal husbandry

All experiments were performed with zebrafish (*Danio rerio*). Zebrafish were maintained, bred and raised at 28^°^C in a light–dark cycle of 14:10 h, respectively according to standard procedures (Westerfield, 2007) and legal regulations (EU Directive 2010_63). Experimental protocols for animal research were approved by governmental authorities of Lower Saxony, LAVES, (AZ33.19-42502-04-22/00208). No selection criteria were used to discriminate between sexes of zebrafish larvae. Embryos were incubated at 28^°^C in 30% Danieau [0.12 mM MgSO_4_, 0.21 mM KCl, 0.18 mM Ca(NO_3_)_2_, 17.4 mM NaCl, 1.5 mM HEPES, pH 7.2]. To prevent pigmentation, at 10–12 h post fertilization (hpf) the medium was exchanged with 30% Danieau medium supplemented with 20 0μM 1-phenyl-2-thiourea (PTU, Sigma Aldrich, St. Louis, MO, USA). For *in vivo* Ca^2+^ imaging, PTU concentration was reduced to 75 μM. Following stable transgenic lines were used that have been described previously: Tg(ca8:FMAtagRFP)^*bz*4^ (Matsui et al., 2014), Tg(ca8-E1B:Hso.Arch3-TagRFPT,GCaMP5G)^*bz*5^, Tg(ca8-E1B:FMATagRFP,GCaMP5G)^*bz*6^, Tg(-7.5ca8:GFP)^*bz*12^ (Namikawa et al., 2019), and Tg(ca8-E1B:Hso.Arch3-TagRFPT,Hso.Arch3-TagRFPT)^*bz*15^ (Matsui et al., 2014).

### Optovin induced swimming

For Optovin-induced swimming, larvae were embedded in 1.5% low melting agarose (Laboratorios CONDA, Madrid, Spain). After solidification of agarose, the matrix surrounding the trunk was removed to allow free trunk movements. Subsequently, the larvae were immersed in freshly diluted 10 μM Optovin (MedChem Express LLC, Monmouth Junction, NJ, USA) in 30%Danieau (stock solution: 100 μM Optovin dissolved in DMSO) (Supplementary Figure 1). Three UV light emitting diode (LED) diodes (405–412 nm) were positioned in a row on a microcontroller board (Arduino UNO). The open-source Arduino software was used to program the board that runs on a loop, with an ON-OFF cycle of 5 s of UV light followed by 15 s of darkness (Supplementary Figure 1), at the same time swimming related Ca^2+^ transients were recorded using a confocal microscope. All larvae used were naïve to the Optovin-stimulus and were not use on consecutive days repeatedly.

### CNQX treatment

6-cyano-7-nitroquinoxaline-2,3-dione (CNQX) (Sigma-Aldrich Biochemie GmbH, Hamburg, Germany) was prepared as stock solution (10 mM in water) and kept as aliquots at –20^°^C. Freshly thawed CNQX was diluted to 20 μM in 30% Danieau directly before use.

### Microinjection

For labeling individual PCs, zebrafish larvae were injected at the one-cell stage with 2 nl of a pTol2-2xcpce-EGFP construct (Namikawa et al., 2019) at a concentration of 5–10 ng/μl together with Tol2-encoding mRNA (25 ng/μl) and 0.05% Phenol Red into the yolk. At 3 dpf injected embryos were inspected under a fluorescence stereomicroscope for sparse EGFP fluorescence in the PC layer. Larvae suitable for single cell imaging were collected for further analysis.

### Image acquisition and analysis

Anesthetized larvae (0.02% Tricaine/30% Danieau) were embedded in 1.5% low-melting agarose in imaging chambers with dorsal side facing the coverslip overlaid with 30% Danieau/0.02% Tricaine, whereas for recording Ca^2+^-dynamics Tricaine was omitted. All image recordings were performed using a confocal laser scanning microscope (TCS SP8, Leica Microsystems, Wetzlar, Germany) at 28^°^C and rendered using the Las-X software (Leica Microsystems, Wetzlar, Germany).

For *in vivo* Ca^2+^ imaging combined with Optovin triggered swimming, short confocal movies about 2 min each were acquired with a frame interval of 500 ms. GCaMP5G fluorescence was recorded across the PC layer at a scan rate of two frames/s with optical sections of 10.92 μm using a 20x water immersion objective (NA: 0.75).

Raw data of Ca^2+^ transient recordings were processed with the help of ImageJ (NIH, Bethesda, MD, USA) plugin descriptor-based series registration (2d/3d+t). Within the Purkinje cell layer three regions of interests (ROIs) as described by Matsui et al. (2014) were defined. To measure ?F/F0, the first 10 frames, during which larvae were not stimulated with UV-light, were used to determine the average intensity (F0), which corresponds to the resting state that was used for processing the remaining frames. Ratiometric analysis of GCaMP5G fluorescence in relation to tagRFP-T intensities revealed (?F). These mean fluorescence intensities of GCaMP5G within ROIs at each time point were subtracted and divided by the average fluorescence intensity of frames (?F/F0). Only increases in signal intensity above 1.5 were included in the analysis to discriminate Ca^2+^-signals from background activity.

Purkinje cell morphology was recorded using a 63x water immersion objective (NA1.4) and optical sectioning of 1.07 μm slices at increments of 0.36 μm. For spine density analysis dendritic protrusions through the z-planes of individual PC 3D reconstructions were counted using the Fiji Plugin Multi Point. Linear membrane protrusions longer than 1.9 μm were considered filopodia according to Peters and Kaiserman-Abramof (1970) and excluded from spine counts. Spine density was quantified as number of spines per length of the dendritic branch in μm.

Graphs and tables were generated using Microsoft Excel (Microsoft Corporation, Redmond, WA, USA) or Prism 6 (GraphPad Software Inc, San Diego, CA, USA).

### Electrophysiology

Purkinje cells were recorded from zebrafish larvae at 5 dpf. Larvae were immobilized in 30% Danieau with 10 μM D-Tubocurarine and embedded in 2% low-melting agarose (Carl Roth, Karlsruhe, Germany) in extracellular solution. Tissue covering the cerebellum was removed with a fine glass capillary under stereo microscopic control (Schramm et al., 2021). Agarose blocks with fish inside were glued to 15 mm coverslips and transferred into a recording chamber perfused with standard extracellular solution constantly bubbled with carbogen gas. Viability of the animals was regularly monitored for by visual inspection of blood circulation. Cells were visualized with a 40x LUMPlanFL N water immersion objective (NA: 0.80, Olympus, Germany) on a SliceScope (Scientifica, Uckfield, United Kingdom) equipped with infrared optics and filters for the detection of FITC (visualization of cells) and tagRFPT (without excitation filter for detection of Alexa 594 and optogenetic stimulation, Chroma Technology GmbH, Olching, Germany). A Polychrome V monochromator (FEI Munich GmbH, Gräfelfing, Germany) was used for illumination and optogenetic stimulation. An ELC-03XS amplifier (NPI Electronic GmbH, Tamm, Germany), an ITC-18 interface and Patchmaster software (both HEKA Elektronik GmbH, Lambrecht, Germany) were used for whole-cell and cell-attached voltage clamp recordings and data acquisition. Electrophysiological recordings were acquired at a sampling rate of 20 kHz after current filtering at 1.3 kHz. Patch pipettes, made from borosilicate glass (Kwik-FIL glass capillaries, OD: 1.5 mm, #1B150F-4, World Precision Instruments GmbH, Friedberg, Germany), had resistances of 7–14 MΩ when filled with the intracellular solution containing (in mM): K-gluconate (115), KCl (15), MgCl_2_ (2), EGTA (10), Mg-ATP (4), and HEPES (10), pH 7.2 (KOH) supplemented with 100 μM Alexa594 hydrazide (Molecular Probes, Eugene, OR, USA) to confirm cell morphology. The standard extracellular solution contained (in mM): NaCl (134), KCl (2.9), MgCl_2_ (1.2), CaCl_2_ (2.1), HEPES (10), and glucose (10), pH 7.8 (NaOH). Purkinje cells were identified by their EGFP or GCaMP5G fluorescence using an excitation wavelength of 480 nm. Pictures were recorded using Live Acquisition Software (FEI Munich GmbH, Gräfelfing, Germany) and a digital Orca R2 camera (Hamamatsu Photonics, Hamamatsu, Japan).

For the approach with the recording pipette a digital zoom factor of 2x was used. PCs were clamped at a holding potential of –60 mV. All voltage clamp data were recorded in standard extracellular solution. This solution was supplemented with CNQX (20 μM, Sigma-Aldrich Biochemie GmbH, Hamburg, Germany), where indicated. For optogenetic stimulation of Arch3 a wavelength of 517 nm was set using Live Acquisition software (FEI Munich GmbH, Gräfelfing, Germany) and light was applied for 30–60 s during voltage clamp recordings. Using a digital power meter (PM100D, Thorlabs GmbH, Bergkirchen, Germany) the power of the illuminating light was measured and lay at around 0.3 mW (0.45 mW/cm^2^). Action potential quantification was performed before (control) and during phases of photostimulation.

### Statistical analysis

For plotting the graphs and performing the appropriate statistical tests, the data sets were imported into Prism 6 (GraphPad Software Inc, San Diego, CA, USA). Unless otherwise mentioned, all data in the graphs are presented as mean + SD.

For statistical analysis the obtained data were subjected to normality test using D’Agostino-Pearson Omnibus (K2). For comparing more than two groups, a one-way ANOVA followed by Tukey’s multiple comparisons test for normally distributed data was applied, else ANOVA Kruskal-Wallis test followed by Dunn’s multiple comparisons test was used.

For comparing only two groups, the data were tested for normality using D’Agostino-Pearson Omnibus (K2), F-test was used for comparing variances. A two-tailed unpaired *t*-test was applied for normally distributed data with equal variances, whereas for normally distributed data with unequal variances two-tailed unpaired *t*-test with Welch’s correction was used for comparing two different groups. Additionally, for the data that did not pass the normality test two-tailed Mann-Whitney test was applied.

For statistical analysis of obtained electrophysiological results data were subjected to Shapiro-Wilk test for normality. If normality could be assumed, a one-way ANOVA with post-hoc Tukey test was performed, otherwise data were subjected to a non-parametric Mann-Whitney rank sum test.

## Results

### Establishment of functional domains in the zebrafish cerebellar PC population

**FIGURE 1:**
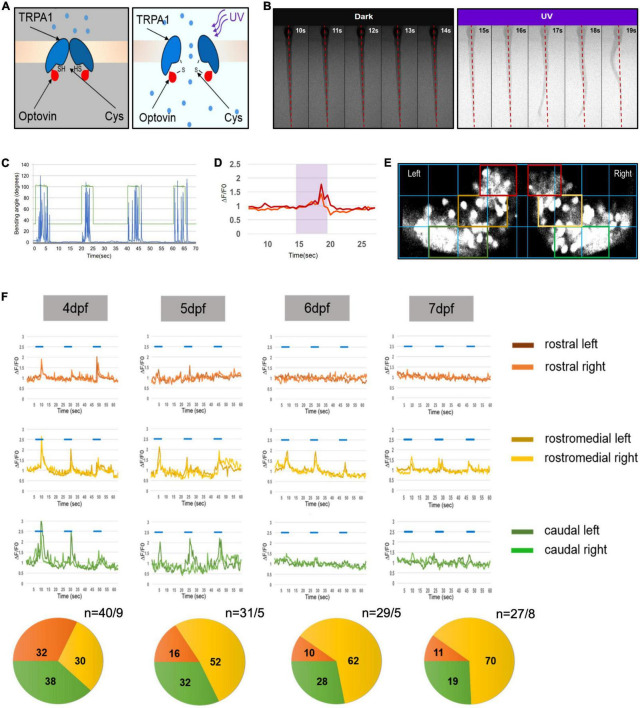
Progressive development of functional domains in the zebrafish Purkinje cell (PC) population during cerebellar development. **(A)** Graphic illustration of transient receptor potential A1 (TrpA1) channel activation in sensory neurons of the trunk by UV illumination of Optovin. **(B)** Montage of consecutive frames of 1 s intervals showing deflection of trunk in the presence of Optovin with and without UV light (red dashed line marks dorsal midline during resting state). **(C)** Graph illustrating angle of bending of trunk (blue peaks) during the time UV light was on (peaks in green) and off (green line). **(D)** Examples of Ca^2+^ transient amplitudes (red and orange) during Optovin induced swimming upon UV illumination (period marked in turquoise). **(E)** The grid used to define rostral (red boxes), rostromedial (yellow boxes) and caudal territories (green boxes) in the zebrafish PC layer expressing the calcium indicator GCaMP5G. **(F)** Illustration of measured swimming correlated Ca^2+^ transients (?F/F0) of different regions of PC layer from 4 to 7 dpf (blue lines indicate periods of UV-illumination). Pie charts below display the percentage of swimming-correlated Ca^2+^-transients within the different PC regions with respect to days of analysis. To the right of pie charts *n* indicates number of recorded transients/number of larvae.

To investigate the development of a functional regionalization in the zebrafish cerebellum a behavioral assay is needed that triggers PC activity prior to the regionalization process. Previous studies had demonstrated that UV-illumination triggers an early escape behavior in zebrafish larvae (Guggiana-Nilo and Engert, 2016). But induction of swimming by UV-illumination alone was only partly reliable and not predictable at 4 days post fertilization (dpf) due to feeble movements during these developmental stages. It has been shown that the photosensitive compound Optovin activates upon UV illumination early differentiating sensory neurons expressing the receptor A1 of the transient receptor potential A1 (TrpA1) receptor family including those neurons in the trunk (Figure 1A). This triggers a motor reflex, enabling light-controlled trunk movements for the duration of UV-illumination in larvae as young as 30 h post fertilization (hpf) (Kokel et al., 2013; Lam et al., 2017). Indeed, UV-illumination in the presence of 10 μM Optovin triggered swimming-related trunk movements paired with the UV-stimulus in a predictable and controlled pattern already at 4 dpf (Figures 1B, C).

To test whether Optovin-induced swimming results in increased Ca^2+^ transients in PCs, we recorded swimming-correlated Ca^2+^ transients of larvae of the stable transgenic strain Tg(ca8-E1B:FMATagRFP,GCaMP5G)^*bz*6^. Zebrafish larvae of this strain express the genetically encoded Ca^2+^-indicator (GECI) GCaMP5G specifically in PCs (Matsui et al., 2014). Such GECIs do not resolve individual events of neuronal activity, but report a long-lasting cumulative activity of neurons, which is well-suited to identify groups of neurons at the population level that are responsive to a certain stimulus. Optovin-triggered swimming movements resulted in an upregulation of GCaMP5G-fluorescence in a temporally restricted manner reflecting periods of trunk and tail deflections associated with swimming (Figure 1D).

To next address the development of functional domains in the PC layer, we overlaid images of GCaMP5G fluorescence recorded during Optovin-induced swimming with the same grid described by Matsui et al. (2014) to define rostral, rostromedial and caudal regions (Figure 1E). The analysis of recorded Ca^2+^-transients in the PC layer associated with swimming movements at 4 dpf showed that the total percentage of swimming-correlated Ca^2+^-transients were nearly equally distributed among all PC regions (Figure 1F, rostral: 32%, rostromedial: 38%, caudal: 30%). These findings suggest that PCs from all regions of the PC layer are physiologically involved in this behavior with similar probability, indicating that the PC layer at 4 dpf is not regionalized in its Ca^2+^-activity with respect to this behavior.

When these measurements were repeated on consecutive days the maximum percentage of Ca^2+^ transients were progressively confined to the rostromedial region increasing from 30% at 4 dpf to 70% at 7 dpf, with a decline in responsiveness in rostral and caudal regions (Figure 1F). These observations indicate that by 6 dpf the PC layer displays a clear regionalized response to Optovin-stimulated trunk movements.

### Changes in dendritic spine density correlate with progressive functional regionalization

**FIGURE 2:**
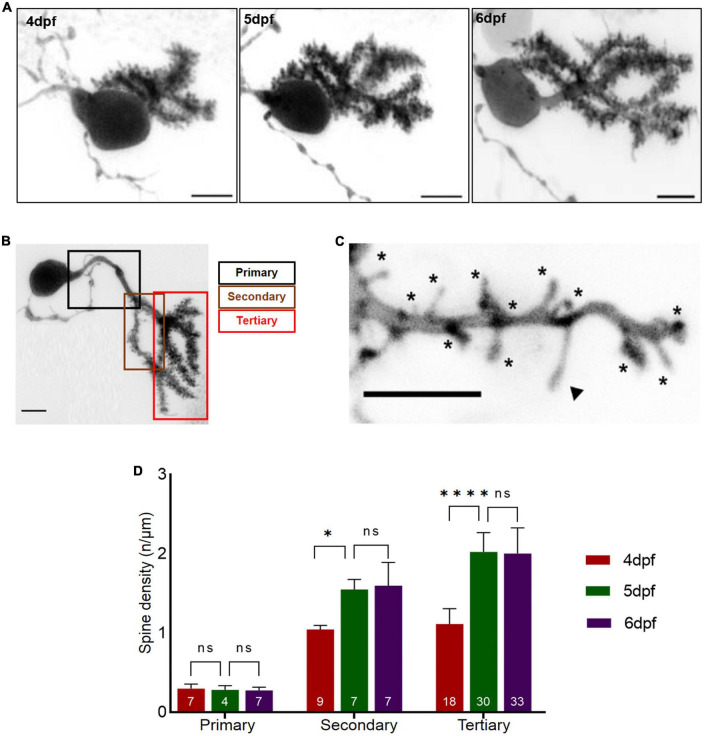
Dendritic spine density of individual Purkinje cell (PCs) with respect to developmental age of larvae. **(A)** Example of a 3D-reconstructed single PC from 4 to 6 dpf recorded by high-resolution confocal microscopy. Scale bar: 5 μm. **(B)** Single PC with demarcated dendritic branches. Scale bar: 5 μm. **(C)** Higher magnification of dendritic branch with visualized protrusions (asterisks indicate spines included for counting; arrowheads mark filopodia structures excluded from counting). Scale bar: 5 μm. **(D)**. The graph displays spine density along primary, secondary and tertiary dendritic branches from 4 to 6 dpf larvae. The data are represented as mean + SD. Numbers inside the bars include total number of dendritic branches analyzed of different individual PCs. Ordinary one-way ANNOVA followed by Tukey’s multiple comparisons test was applied for normally distributed data, else Kruskal-Wallis test followed by Dunn’s multiple comparisons test was used in **(D)**. Denotations for significance are non-significant (n s) *p* > 0.1234, *p < 0.0332, ^****^p < 0.0001.

The density of spines along dendritic branches can be used to evaluate the maturity of differentiating neurons. At 4 dpf, PC primary dendrite specification is completed and major dendritic branches are established (Tanabe et al., 2010). We therefore wondered, if formation of PC dendritic spines shows a developmental pattern similar to the progressive specification of functional PC domains. Mosaic labeling of single PCs was achieved by microinjecting a plasmid expressing membrane targeted EGFP under control of the PC specific enhancer cpce (Namikawa et al., 2019) into Tg(ca8:FMAtagRFP)^*bz*4^ embryos (Matsui et al., 2014) at the one cell stage. At 3 dpf zebrafish larvae with individual fluorescent PCs labeled by EGFP expression were selected. During the consecutive days from 4 to 6 dpf high resolution confocal microscopy was used to visualize spines along different dendritic branches from the same PCs (Figures 2A–C). The primary, secondary and tertiary dendritic structures of these PCs were manually traced and spine density was quantified along the dendritic branches of individual PCs from 4 to 6 dpf (Figure 2D).

At 4 dpf a large number of spines could be observed along all dendritic branches with exception of the primary branch. Quantification revealed that primary PC dendrites contained only few spines, and dendritic spine density remained constant from 4 to 6 dpf (Figure 2D, ordinary one-way ANOVA, *F*_2, 15_ = 0.3638, *p* = 0.7010, 4 dpf: *n* = 7, 0.30 ± 0.057; 5 dpf: *n* = 4, 0.28 ± 0.053; 6 dpf: *n* = 7, 0.28 ± 0.040; *n* indicates number of analyzed dendritic branches). In contrast, spine density along secondary and tertiary branches increased with developmental age of larvae, with tertiary dendrites displaying a higher spine density compared to secondary dendrites from 4 to 6 dpf (Figure 2D, secondary dendrites: ANOVA, *p* = 0.0004, 4 dpf: *n* = 9, 1 ± 0.053; 5 dpf: *n* = 7, 1.6 ± 0.13; 6 dpf: *n* = 7, 1.6 ± 0.29, tertiary dendrites: ANOVA, *p* < 0.0001, 4 dpf: *n* = 18, 1.1 ± 0.19; 5 dpf: *n* = 30 2.0 ± 0.24; 6 dpf: *n* = 33, 2.0 ± 0.32). Interestingly, spine density along secondary and tertiary dendrites increased immensely from 4 to 5 dpf (Figure 2D, secondary dendrites: ANOVA, adjusted *p*-value = 0.0100, 4 dpf: *n* = 9, 1.0 ± 0.0.053; 5 dpf: *n* = 7, 1.6 ± 0.1; tertiary dendrites: ANOVA, adjusted *p*-value < 0.0001, 4 dpf: *n* = 18, 1.1 ± 0.19; 5 dpf: *n* = 30, 2 ± 0.24), but remained constant from 5 to 6 dpf (Figure 2D, secondary dendrites: ANOVA, adjusted *p*-value > 0.9999, 5 dpf: *n* = 7, 1.6 ± 0.13; 6 dpf: *n* = 7, 1.6 ± 0.29, tertiary dendrites: ANOVA, adjusted *p*-value > 0.9999, 5 dpf: *n* = 30, 2 ± 0.24; 6 dpf: *n* = 33, 2.0 ± 0.32).

In summary, onset of PC differentiation at 3 dpf by initiation of dendritogenesis (Tanabe et al., 2010) followed by the appearance of a significant number of spines at 4 dpf suggests the initiation of synaptogenesis. This is consistent with the observation of first occurring Ca^2+^-signals at 4 dpf in PCs during Optovin-stimulated trunk movements (Figure 1F). Subsequent maturation and refinement of dendrites lasts until 6dpf, when a plateau in spine density is reached. This developmental time window of PC maturation correlates well with the observed time course of progressive functional regionalization in the PC layer.

### Silencing of PC activity reduces dendritic spine density

Since dendritic spine density is regulated by neuronal activity (McAllister, 2000; Segal, 2010), we wondered whether functional regionalization in the PC layer is influenced by PC activity itself. Purkinje cell excitability in zebrafish is mediated by AMPA-receptors (Sengupta and Thirumalai, 2015), hence we used 6-cyano-7-nitroquinoxaline-2,3-dione (CNQX) as AMPA-receptor antagonist in bath incubation experiments (Pietri et al., 2009; Theisen et al., 2018) and performed direct electrophysiological recordings from PCs (*n* = 9) *in vivo* at 5 dpf. Bath application of 20 μM CNQX significantly reduced spontaneous postsynaptic currents (sPSC) almost completely silencing PC activity (Supplementary Figure 2) and confirming that CNQX is able to suppress PC activity in zebrafish larvae.

**FIGURE 3:**
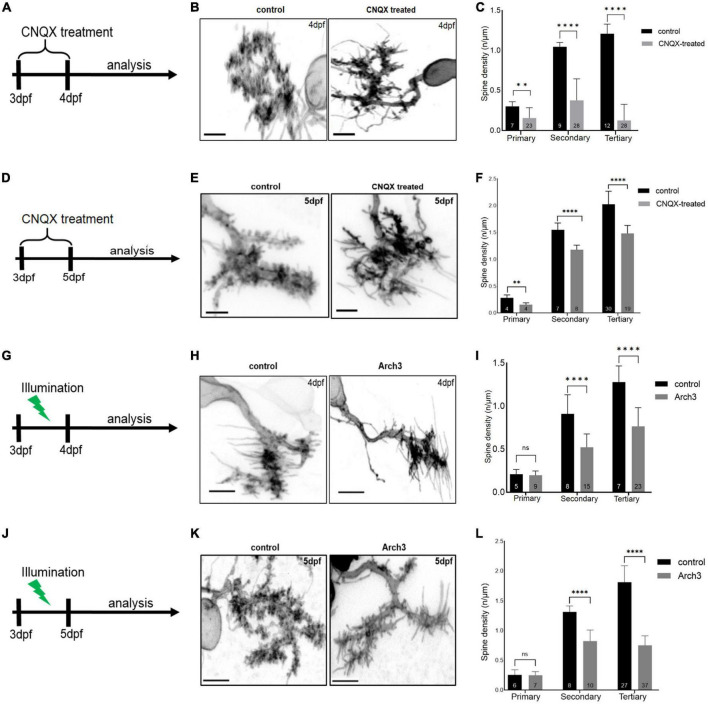
Inhibiting Purkinje cell (PC) neuronal activity reduces PC spine density. Schematic drawing illustrating the scheme of 6-cyano-7-nitroquinoxaline-2, 3-dione (CNQX) treatment from 3 dpf to analysis on 4 dpf **(A)** and on 5 dpf **(D)**. Maximum intensity projection of untreated and CNQX treated PC at 4 dpf **(B)** and 5 dpf **(E)**. Scale bar: 5 μm. Representation of spine density on primary, secondary and tertiary branches of CNQX treated PCs (gray) vs. controls (black) at 4 dpf **(C)** and at 5 dpf **(F)**. Schematic drawing illustrating the start of pulsed illumination of 517 nm monochromatic light until the confocal microscopy analysis of Arch3 and non-Arch3 expressing larvae at 4 dpf **(G)** and 5 dpf **(J)**. Maximum intensity projection of PC at 4 dpf **(H)** and 5 dpf **(K)** from Arch3 larvae and non-Arch3 expressing larvae, respectively. Scale bar: 5 μm. Graph showing PC spine density quantification in 4 dpf larvae **(I)** and 5 dpf larvae **(L)** on primary, secondary and tertiary dendritic branches of Arch3 larvae (gray) vs. controls (black). The data in graphs are presented as mean + SD. Numbers inside the bars include total number of dendritic branches analyzed of different individual PCs. For **(C)**, **(F)**, **(I)**, **(L)** two-tailed unpaired *t*-test for normally distributed data with equal variances was used, for normally distributed data with unequal variances two-tailed unpaired *t*-test with Welch’s correction was applied. Additionally, for the data that did not pass the normality test, two tailed Mann-Whitney test was applied. Denotations for significance are non-significant (n s) *p* > 0.1234, ^**^p < 0.0021, ^****^p < 0.0001.

Next, we tested the role of neuronal activity in modulating dendritic spine density. For this purpose, larvae with PCs labeled by membrane targeted EGFP expression in a sparse, mosaic manner were selected at 3 dpf and treated with 20 μM CNQX for 24 or 48 h, respectively (Figures 3A, D). Untreated larvae were used as controls. Individual PCs of both groups were subjected to high resolution imaging at 4 dpf or 5 dpf by laser scanning confocal microscopy (Figures 3B, E). This analysis revealed that spine density of activity-silenced PCs treated with CNQX were significantly decreased after 24 h of CNQX-treatment compared to controls along primary (Figure 3C, control: *n* = 7, 0.30 ± 0.057; CNQX-treated: *n* = 23, 0.16 ± 0.13; two-tailed Mann Whitney test, *p* = 0.0011), secondary (Figure 3C, control: *n* = 9, 1.0 ± 0.053; CNQX-treated: *n* = 28, 0.38 ± 0.27; two-tailed unpaired *t*-test with Welch’s correction, *p* < 0.0001) as well as tertiary dendrites (Figure 3C, control: *n* = 12, 1.2 ± 0.12, CNQX-treated: *n* = 28, 0.13 ± 0.20; two-tailed Mann Whitney test, *p* < 0.0001).

Similarly, a reduction in spine density was observed after 48 h of CNQX-mediated silencing of activity at 5 dpf compared to controls along primary (Figure 3F, control: *n* = 4, 0.28 ± 0.053; CNQX-treated: *n* = 4, 0.15 ± 0.035; two-tailed unpaired *t*-test, *p* = 0.0069), secondary (Figure 3F, control: *n* = 7, 1.55 ± 0.128; CNQX-treated: *n* = 8, 1.18 ± 0.088; two-tailed unpaired *t*-test, *p* < 0.0001) as well as tertiary dendrites (Figure 3F, control: *n* = 30, 2.02 ± 0.244, CNQX-treated: *n* = 19, 1.48 ± 0.149; two-tailed unpaired *t*-test with Welch’s correction, *p* < 0.0001).

To corroborate these findings by cell type specific silencing of PC activity, we selected long-term optogenetics using Archaerhodopsin3 (Arch3) as light-gated hyperpolarizing outward-directed proton pump (Chow et al., 2010). We built a custom illumination chamber for petri dishes (diameter of 6 cm) equipped with an LED-panel that generates pulsed illumination of 517 nm monochromatic light (0.37, 0.52 mW/cm^2^) at 1 Hz (duration of illumination 120 ms) to not exhaust light-gated Arch3 proton efflux (Supplementary Figures 3A, B). To confirm that this setup inhibits PC activity, we first performed direct electrophysiological recordings from individual PCs (*n* = 5) at 5 dpf of the Tg(ca8-E1B:Hso.Arch3-TagRFPT,GCaMP5G)^*bz*5^ transgenic line (Matsui et al., 2014), while Tg(ca8-E1B:FMATagRFP,GCaMP5G)^*bz*6^ were used as control. Equivalent illumination conditions (Supplementary Figure 3C) using a monochromator during electrophysiological recordings eliminated spontaneous postsynaptic currents (sPSC) in PCs silencing their activity nearly completely and confirming that Arch3 is efficiently stimulated under this illumination paradigm (Supplementary Figure 4).

To ensure even more efficient Arch3-mediated PC silencing, we used a bidirectional Tg(ca8-E1B:Hso.Arch3-TagRFPT,Hso.Arch3-TagRFPT)^*bz*15^ transgenic line (Matsui et al., 2014) expressing two copies of the Arch3-TagRFP-T fusion protein in cerebellar Purkinje cells. Embryos were injected at the one cell stage with pTol2-2xcpce-EGFP. At 3 dpf heterozygous carriers of the Arch3-expressing transgene and sparse mosaic membrane targeted EGPF expression were selected and raised under continuous pulsed illumination for optogenetic PC inhibition for 24 (Figure 3G) or 48 h (Figure 3J), respectively, while Arch3 non-expressing siblings served as control. High resolution confocal microscopy of individual PCs at 4 and 5 dpf (Figures 3H, K) revealed that their spine density in Arch3 expressing larvae after 24 h of illumination along secondary [Figure 3I, control: *n* = 8, 0.91 ± 0.22; Arch3: *n* = 15, 0.52 ± 0.15; two-tailed unpaired *t*-test, *p* < 0.0001, t (5) = 21] and tertiary (Figure 3I, control: *n* = 7, 1.30 ± 0.19; Arch3: *n* = 23, 0.77 ± 0.22; two-tailed Mann-Whitney test, *p* < 0.0001) dendrites was significantly reduced compared to controls.

Similarly, confocal microscopy after 48 h of Arch3 stimulation by light revealed that at 5 dpf the spine density in individual PCs along secondary [Figure 3L, control: *n* = 8, 1.31 ± 0.101; Arch3: *n* = 10, 0.83 ± 0.184; two-tailed unpaired *t*-test, *p* < 0.0001, t (7) = 16] and tertiary [Figure 3L, control: *n* = 27, 1.09 ± 0.278; Arch3: *n* = 37, 0.88 ± 0.294; two-tailed unpaired *t*-test, *p* < 0.0001 t (13) = 62)] dendrites was significantly reduced compared to controls.

Taken together, pharmacological as well as cell-type specific optogenetic silencing of PC- neuronal activity consistently demonstrate that inhibition of neuronal activity in PCs leads to reduction in dendritic spine coverage. Hence the maturation of zebrafish cerebellar PCs is dependent on their proper electrophysiological activity.

### Inhibition of neuronal activity impairs functional regionalization

**FIGURE 4:**
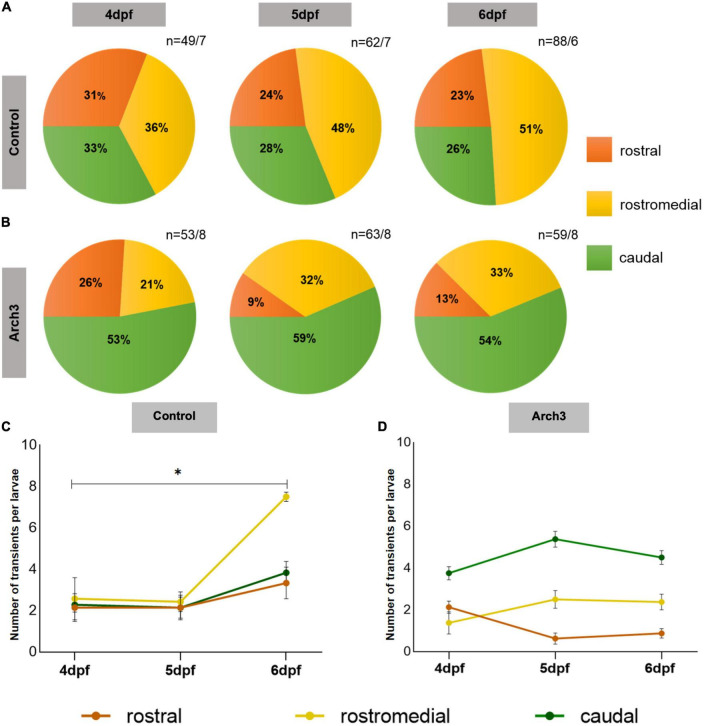
Formation of functional domains in the Purkinje cell (PC) population is impaired upon inhibiting PC neuronal activity. The pie charts represent the percentage of Optovin-induced swimming-correlated Ca^2+^ transients from 4 to 6 dpf localized in different regions of the PC layer. Ca^2+^ transients were recorded after continuous Arch3-stimulating illumination to suppress PC activity in **(A)** Tg(ca8-E1B:FMATagRFP,GCaMP5G)^bz6^ larvae as control and **(B)** Arch3-expressing Tg(ca8-E1B:Hso.Arch3-TagRFPT,GCaMP5G)^bz5^ larvae. *n* represents number of recorded Ca^2+^ transients/number of larvae. The number of transients per larvae in each PC subregion in **(C)** control and **(D)** Arch3-expressing larvae is displayed for consecutive days during which PC maturation occurs. The data are represented as mean ± SEM. Kruskal-Wallis test followed by Dunn’s multiple comparisons test was used in **(C)**. Denotations for significance is *p < 0.0332.

Our results so far demonstrate that development of function specific domains of Ca^2+^ activity in the PC population correlates with formation of dendritic spines and also spine density is dependent on neuronal activity. Therefore, we asked if neuronal activity also modulates functional regionalization. To answer this, we combined optogenetics with Optovin-stimulated swimming assays. Here, larvae of stable transgenic lines Tg(ca8-E1B:Hso.Arch3-TagRFPT,GCaMP5G)^*bz*5^ and Tg(ca8-E1B:FMATagRFP,GCaMP5G)^*bz*6^ as controls, were raised under pulsed illumination from 3 dpf until swimming correlated Ca^2+^ transient recordings were performed. At 4 dpf Ca^2+^ transients were nearly equally distributed among PC regions (Figure 4A, 4 dpf: rostral 31%, rostromedial 37%, and caudal 33%) in control larvae. As observed previously, this pattern changed during the following 2 days and Ca^2+^ transients during Optovin stimulated swimming became progressively confined to the rostromedial region of the PC population (Figure 4A, 6 dpf: rostral 23%, rostromedial 51%, and caudal 26%). Thus, continuous exposure to pulsed illumination did not interfere with proper functional regionalization of PCs (compare Figures 1F, 4A). This rostromedial region has been identified previously with the help of PC-specific Arch3 optogenetic inhibition to coordinate proper execution of oriented swimming (Matsui et al., 2014).

When Ca^2+^ transients per larvae were displayed for each PC region an overall increase of these transients was observed for each region from 4 to 6 dpf (Figure 4C) corresponding to the increase in spine density. Yet, Ca^2+^ transients in the rostromedial PC region were the only to show a statistically significant elevation from 4 to 6 dpf (Figure 4C, rostromedial: ANOVA, adjusted *p*-value = 0.0124, 4 dpf: 2.6 ± 1.02, *n* = 7; 6 dpf: 7.5 ± 0.23, *n* = 6; *n* indicates number of larvae) in comparison to the rostral and caudal PC regions suggesting that during the course of regionalization the rostromedial domain becomes more sensitive in responding to Optovin-induced trunk movements compared to the other subregions.

In Arch3-expressing larvae with optogenetic inhibition of neuronal activity, maximum percentage of measured Ca^2+^ transients were localized in the caudal region already at 4 dpf (Figure 4B, dpf: rostral 26%, rostromedial 21%, and caudal 53%). This pattern is different from larvae at 4 dpf with actively firing PCs (Figure 1F), and likely reflects altered initial PC circuitry formation under conditions of impaired spontaneous PC activity. Importantly, when Ca^2+^ measurements were performed on the following days after continuously silencing PC activity, the pattern of swimming-correlated Ca^2+^-transients did not change dramatically, but was clearly different from controls. Optovin-stimulation mediated Ca^2+^-transients appeared mostly in the caudal and not in the rostromedial region of the PC population and remained nearly stagnant in their percentage from 4 to 6 dpf (Figure 4B, caudal: 4 dfp: 53%, 6 dpf: 54%). In the rostromedial region some increase in the response to Optovin-stimulation from 21 to 33% could be observed from 4 to 6 dpf, suggesting that this region became more responsive and such slight increase in responsiveness could be observed when Ca^2+^ transients per larvae were displayed for each PC region (Figure 4D, rostromedial: 4 dpf: 1.38 ± 0.53, *n* = 8; 6 dpf: 2.38 ± 0.38, *n* = 8). Yet, this increase did not reach levels of controls, in which more than half of the Optovin-stimulation elicited Ca^2+^-transients appeared in the rostromedial region. In the rostralmost region a decrease from 26 to 13% in responsiveness could be observed corresponding to the moderate increase in the rostromedial region. Of note, in none of the domains including the most responsive caudal one (6 dpf: 4.5 ± 0.31 transients per larva, *n* = 8 larvae) the number of Ca^2+^ transients per larvae in each subregion reached levels of responsiveness found in controls in the rostromedial domain (6 dpf: 7.5 ± 0.23 transient per larva, *n* = 6 larvae) (compare Figures 4C, D). Furthermore, in none of these regions the increase or decrease in Ca^2+^ transients per larvae reached statistical significance, supporting the suggestion that Arch3-mediated inhibition of PC-activity results in a lower responsiveness and stagnation of the regionalization pattern reached at 4 dpf.

In conclusion, these findings illustrate that silencing PC activity alters the progressive development of function specific domains in the PC population, initially rendering the caudal most instead of the rostromedial region of PCs more responsive to Optovin-mediated stimuli. While a moderate increase in responsiveness of rostromedial PCs under conditions of constitutive PC silencing was observed during PC maturation, this increase did not enable rostromedial PCs to become the predominant domain in the PC layer responding to Optovin-mediated trunk movement. Furthermore, the process of regionalization in all subregions was interrupted and remained static. These findings suggest that the establishment of functional domains within the PC population is likely mediated by the electrophysiological activity of PCs themselves.

## Discussion

Our results show that zebrafish PCs are not *a priori* confined to a distinct physiological response and functional output, but that they instead progressively refine their response within regional territories of the zebrafish PC layer. This spatially refined response within the PC population occurs during stages of PC maturation and development of new spines suggesting the involvement of neuronal activity in connectivity refinement. Indeed, silencing neuronal activity of PCs not only reduced spine formation, but was paralleled by an altered functional regionalization. This suggests that the segregated organization of the cerebellar PC population with subsets of PCs controlling different behaviors is subjected to a process of development based on physiological activity patterns of PCs.

Currently, we cannot distinguish whether Ca^2+^ signals in the PC layer upon Optovin-stimulated trunk movements represent either processing of sensory information, motor kinematics or motor intent. Nevertheless, our studies could reveal that Optovin-stimulation leads to a progressively regionalized Ca^2+^-activity pattern in the cerebellar PC population, which seems to be mediated by an increase in responsiveness of PCs in the relevant (rostromedial) territory. In a recent elegant study such a regionalized organization of the PC layer in zebrafish was demonstrated for a number of visual and motor activities such as processing of visual sensory information or mediating locomotion. Here, responses of PCs to eye or tail motion were found to rather broadly activate the entire PC population (Knogler et al., 2019). Light-activated swimming behavior by Optovin is not visually mediated, but involves the spinal sensory-motor reflex (Kokel et al., 2013). Thus, PC activity following Optovin-activation likely represents either the processing of sensory information from the spinal cord or activating trunk locomotion. Unambiguous clarification will require in depth electrophysiological studies combined with functional analysis in order to identify the circuitry between activated Optovin-stimulated sensory neurons and cerebellar PCs. Nevertheless, because of the reliability in motor response already during early PC differentiation stages, Optovin-elicited Ca^2+^-transients are well-suited to study the dynamics of the emerging segregation of PC activity in the PC layer associated with swimming.

While in control larvae the rostromedial PC region became more responsive to Optovin-stimulated trunk-movements during stages of PC differentiation, responsiveness to this stimulus remained static and declined in the rostral and caudal PC regions based on percentage. This does not mean that these PC regions are less active in general, but they likely respond to other stimuli as a consequence of functional segregation of the PC population. Further investigations will require the establishment of behavioral assays that elicit Ca^2+^-signals in the PC layer in these areas already before this functional diversification is established. Imaging of Ca^2+^-transients on the population level using genetically encoded Calcium indicators as reporters of cumulative neuronal activity is well-suited to address these questions of regionalization within the PC population. It would be interesting to perform single cell electrophysiology recordings from individual PCs of the different functional PC regions during Optovin-stimulated trunk movements. However, such measurements are invasive and may not allow proper recording during trunk motion due to the triggered movements of the larvae. The advent of voltage-sensitive fluorescent proteins with high temporal resolution and sufficient signal amplitude dynamics may allow for such single cell analytics in the future.

Interestingly, rostral and rostromedial PCs under constantly pulsed green light exposure showed a similar responsiveness to Optovin-stimulation at 4dpf as observed in PCs of control-larvae PCs of wildtype larvae. In contrast, the physiological inhibition of PCs by continuous pulsed light injection resulted in caudal PCs to become already more responsive to the Optovin-stimulus at this early phase of PC maturation compared to other PC regions. Currently, we have no mechanistic explanation for this observation. The data in wild type larvae suggest that PC regionalization is likely mediated by the activity of PCs themselves. One hypothesis could be that inhibition of this physiological activity may allow for a stronger impact of genetic factors regulating PC connectivity, thereby resulting in an altered cerebellar circuitry with caudal PCs contributing more extensively to circuits activated by Optovin-stimulation.

The activity-driven maturation of neuronal connectivity in the cerebellum resembles known mechanisms of neuronal learning and memory formation for example in the mammalian hippocampus (McGaugh, 2000). It will therefore be of interest whether similar cell biological mechanisms and molecular cues mediate the functional regionalization of zebrafish PCs downstream of altered membrane polarization. For example, functional regionalization could be mediated by Ca^2+^ regulated signal transduction factors such as Calmodulin, Ca^2+^/Calmodulin-dependent protein (CaM) kinases, Calcineurin or Calmodulin-binding transcription activators (CAMTAs) (Colomer and Means, 2007). These in turn may drive cytoskeletal rearrangements to promote dendrite maturation and to remove or enhance synaptic contacts to modulate synaptic transmission (Borovac et al., 2018; Shimobayashi and Kapfhammer, 2018). Zebrafish with their accessibility for molecular genetics, *in vivo* imaging and behavioral modulation are well-suited to further expand our understanding about the crucial evolutionary conserved molecular, physiological and functional relationships of neuronal connectivity and plasticity in the central nervous system of vertebrates.

## Data availability statement

The raw data supporting the conclusions of this article will be made available by the authors, without undue reservation.

## Ethics statement

The animal study was reviewed and approved by Niedersächsisches Landesamt für Verbraucherschutz und Lebensmittelsicherheit Postfach 9262 26140 Oldenburg.

## Author contributions

KN, HM, and RK conceived the study and designed experimental approaches. AD, KV, and FH generated and analyzed data. KN, JM, and RK supervised the project. All authors contributed to writing the manuscript and approved the submitted version.

## Supplementary material

Supplementary Figure 1 Arrangement for triggering Optovin induced swimming upon UV illumination. **(A)** Arduino UNO board (left) connected to a power bank via Universal Serial Bus (USB) (not shown in image) and related circuits that are coupled to the breadboard with light emitting diode (LED) diodes of wavelength 405–412 nm (right). **(B)** Drawing showing the setup of Optovin-mediated swimming behavior in larvae. **(C)** Arduino script used to program the broad with ON-OFF time of UV illumination.

Supplementary Figure 2 Bath-application of CNQX inhibits PC activity in zebrafish larvae. **(A)** Schematic drawing of zebrafish head with green fluorescent PCs used for voltage-clamp recordings. **(B)** Tg(-7.5ca8:GFP)^bz12^ (Namikawa et al., 2019) expressing larvae with selective labeling of PCs by green fluorescence at 5 dpf were used for electrode positioning for whole cell-clamp recordings. **(C)** The correct morphology of the recorded cell was verified after electrophysiological recording by dye-filling of patched cells with the red fluorescence dye Alexa 594 followed by verification of colocalization of green and red fluorescence. **(D)** 20 μM CNQX suppressed PC spontaneous postsynaptic currents (sPSC) almost completely [compare example traces of wild type (black colored trace) PC to CNQX-exposed (gray colored trace) PC] in all recordings (*n* = 9, *p* < 0.005 Mann-Whitney rank sum test).

Supplementary Figure 3 Arrangement for optogenetic silencing of Purkinje cells (PCs). **(A)** Custom built illumination chamber with LED panel. **(B)** Set up illustrating generation of pulsed illumination of 517 nm monochromatic light (green color, 0.37, 0.52 mW/cm^2^) that results in stimulating light-gated Arch3 proton efflux. **(C)** Equivalent illumination conditions (0.32, 0.45 mW/cm^2^) were used for electrophysiological recordings to confirm that the delivered optical energy is sufficient to suppress PC activity (Supplementary Figure 4).

Supplementary Figure 4 Illumination of Arch3 expressing Purkinje cells (PCs) suppresses their neuronal activity. **(A)** Tg(Arch-tagRFP-T:PC:GCaMP5G)^bz5^ larvae were used for cell-attached recordings, while larvae were exposed to 517 nm green/yellow light (0.32 mW, continuous illumination for 30–60 s, Supplementary Figure 3) using a monochromator. **(B,C)** The correct morphology of the recorded cell was verified after electrophysiological recording by dye-filling of patched cells with the red fluorescence dye Alexa 594 followed by verification of colocalization of green and red fluorescence. **(D)** Upon 517 nm illumination (horizontal green bar indicates duration of illumination) action potential firing of PCs was transiently almost completely suppressed in Arch3 expressing PCs, (*n* = 5, *p* < 0.05 one way ANOVA with post-hoc Tukey test).
