# Supplementary figures and images for: Functional regionalization of the differentiating cerebellar Purkinje cell population occurs in an activity-dependent manner
Source: Front Mol Neurosci. 2023 Apr 27;16:1166900. doi: 10.3389/fnmol.2023.1166900 (PMC10174242; doi:10.3389/fnmol.2023.1166900)

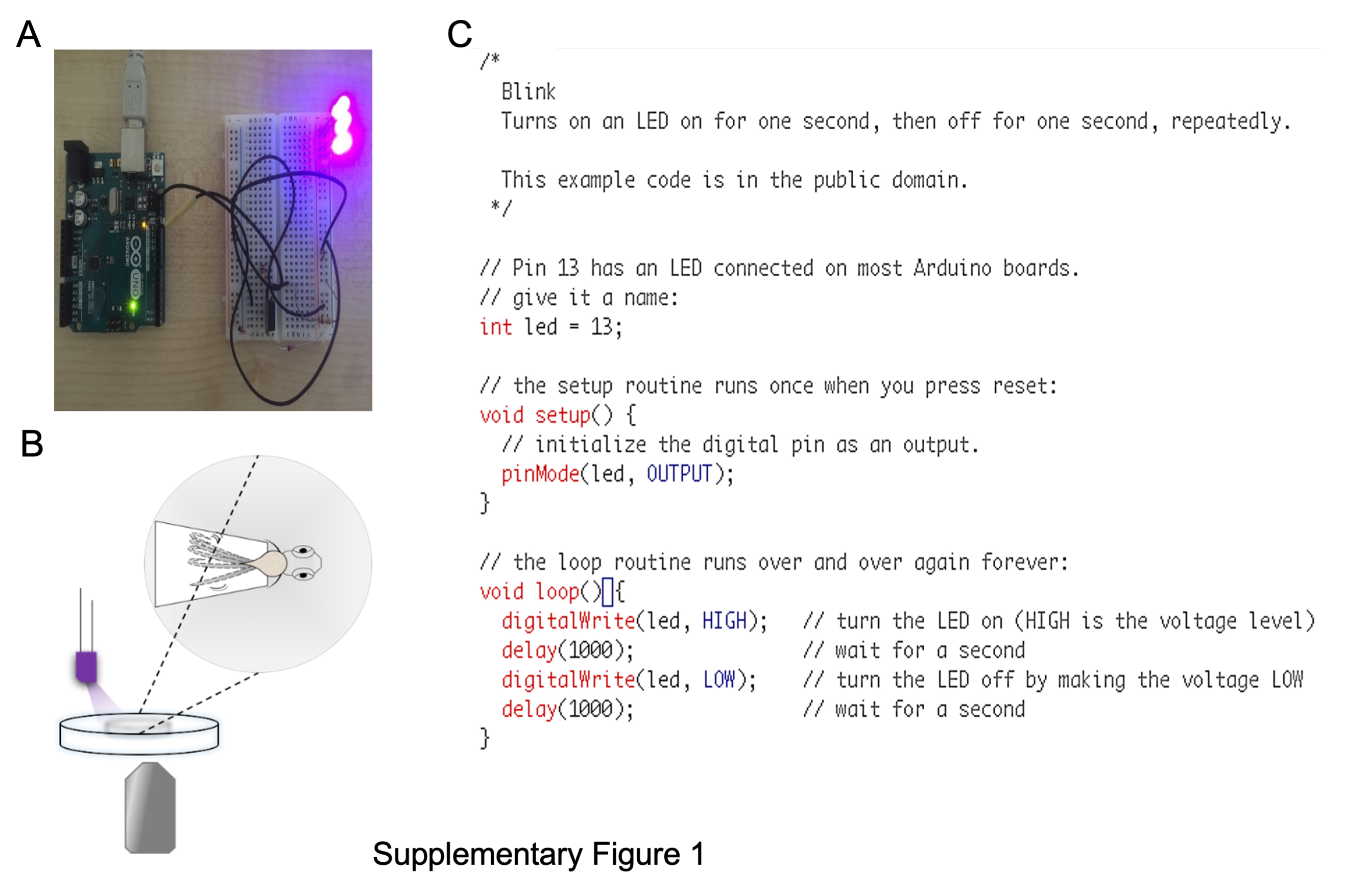

Supplement: Supplementary Figure 1 — Arrangement for triggering Optovin induced swimming upon UV illumination. (A) Arduino UNO board (left) connected to a power bank via Universal Serial Bus (USB) (not shown in image) and related circuits that are coupled to the breadboard with light emitting diode (LED) diodes of wavelength 405–412 nm (right). (B) Drawing showing the setup of Optovin-mediated swimming behavior in larvae. (C) Arduino script used to program the broad with ON-OFF time of UV illumination. [file Image_1.TIF]

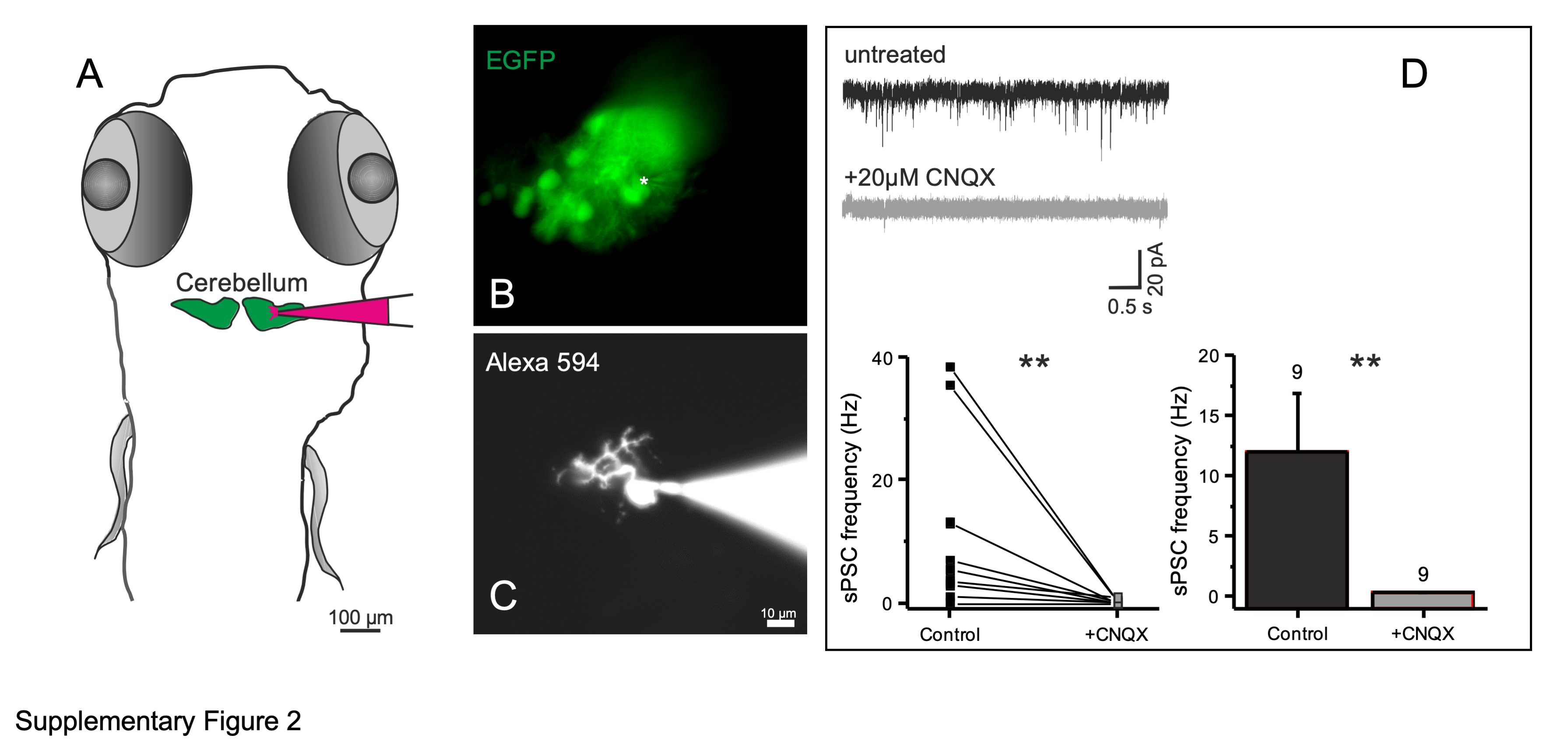

Supplement: Supplementary Figure 2 — Bath-application of CNQX inhibits PC activity in zebrafish larvae. (A) Schematic drawing of zebrafish head with green fluorescent PCs used for voltage-clamp recordings. (B) Tg(-7.5ca8:GFP)bz12 (Namikawa et al., 2019) expressing larvae with selective labeling of PCs by green fluorescence at 5 dpf were used for electrode positioning for whole cell-clamp recordings. (C) The correct morphology of the recorded cell was verified after electrophysiological recording by dye-filling of patched cells with the red fluorescence dye Alexa 594 followed by verification of colocalization of green and red fluorescence. (D) 20 μM CNQX suppressed PC spontaneous postsynaptic currents (sPSC) almost completely [compare example traces of wild type (black colored trace) PC to CNQX-exposed (gray colored trace) PC] in all recordings (n = 9, p < 0.005 Mann-Whitney rank sum test). [file Image_2.TIF]

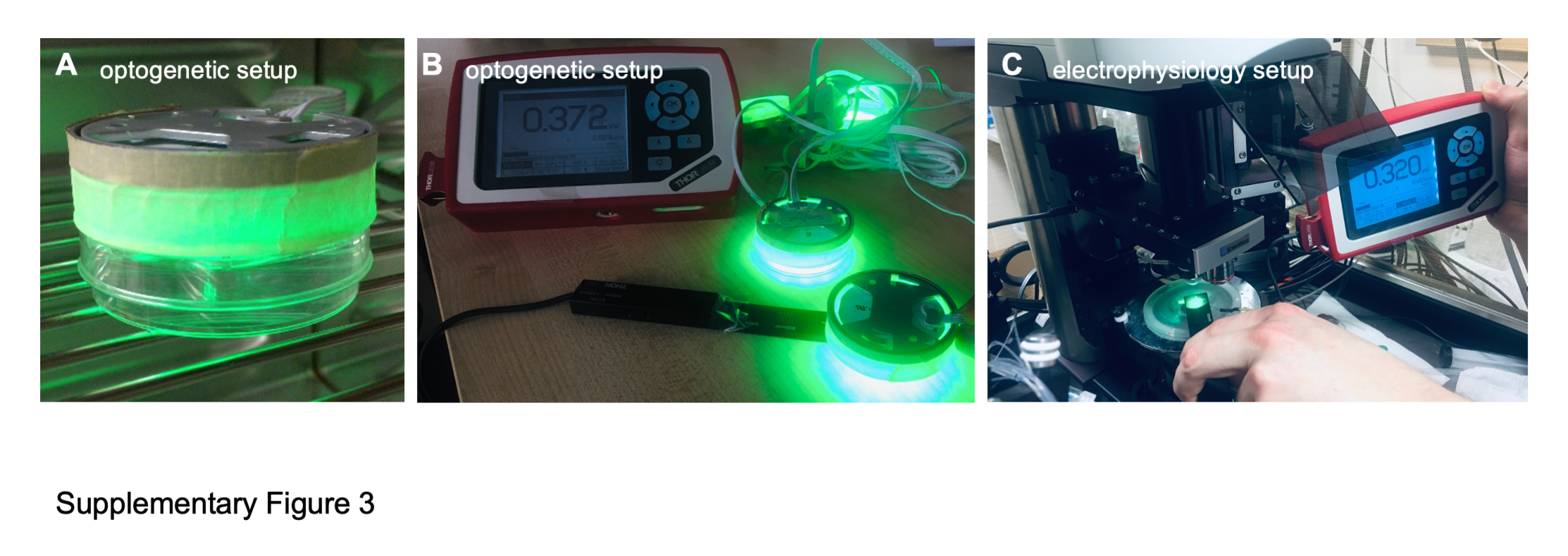

Supplement: Supplementary Figure 3 — Arrangement for optogenetic silencing of Purkinje cells (PCs). (A) Custom built illumination chamber with LED panel. (B) Set up illustrating generation of pulsed illumination of 517 nm monochromatic light (green color, 0.37, 0.52 mW/cm2) that results in stimulating light-gated Arch3 proton efflux. (C) Equivalent illumination conditions (0.32, 0.45 mW/cm2) were used for electrophysiological recordings to confirm that the delivered optical energy is sufficient to suppress PC activity (Supplementary Figure 4). [file Image_3.TIF]

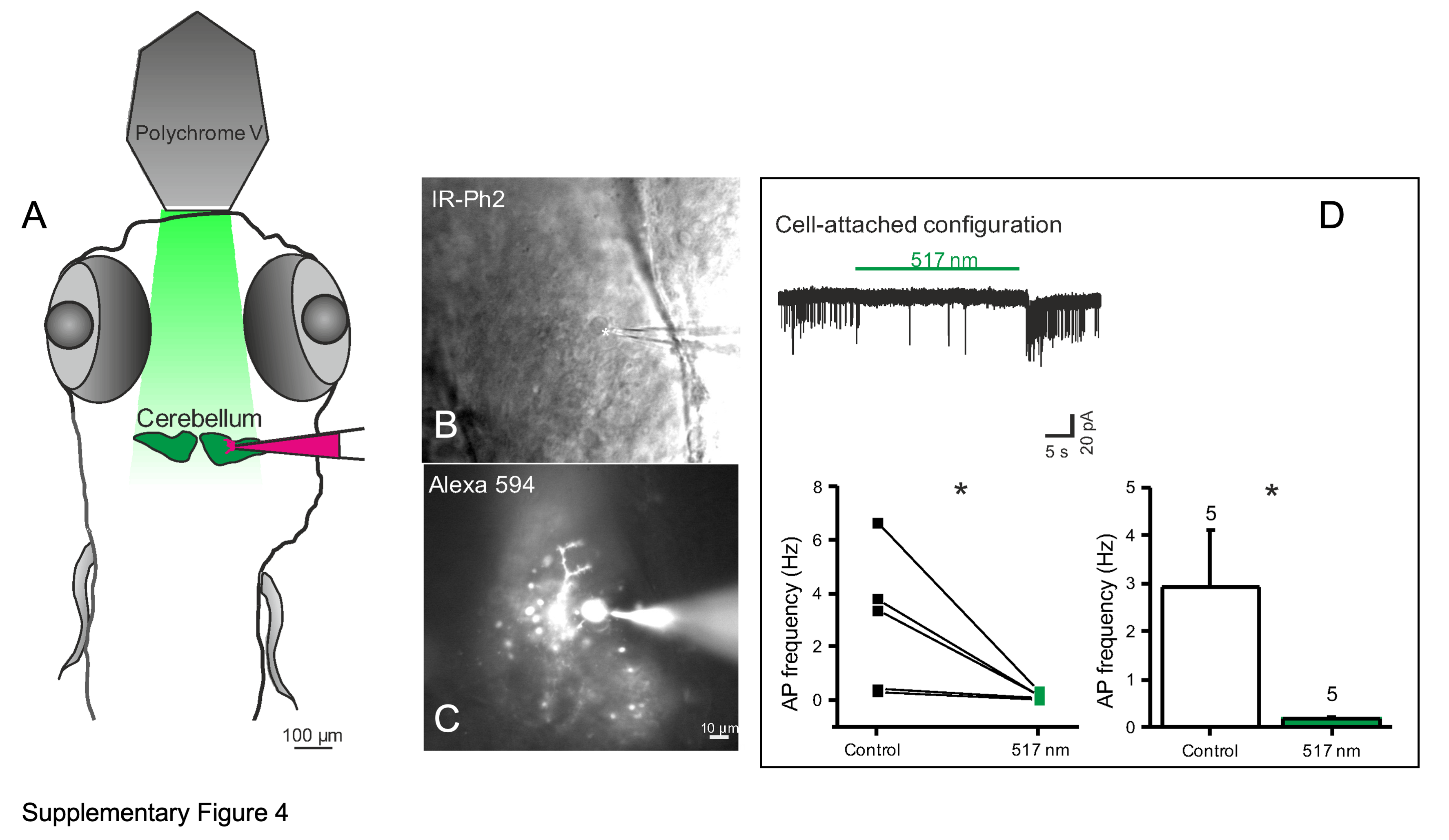

Supplement: Supplementary Figure 4 — Illumination of Arch3 expressing Purkinje cells (PCs) suppresses their neuronal activity. (A) Tg(Arch-tagRFP-T:PC:GCaMP5G)bz5 larvae were used for cell-attached recordings, while larvae were exposed to 517 nm green/yellow light (0.32 mW, continuous illumination for 30–60 s, Supplementary Figure 3) using a monochromator. (B,C) The correct morphology of the recorded cell was verified after electrophysiological recording by dye-filling of patched cells with the red fluorescence dye Alexa 594 followed by verification of colocalization of green and red fluorescence. (D) Upon 517 nm illumination (horizontal green bar indicates duration of illumination) action potential firing of PCs was transiently almost completely suppressed in Arch3 expressing PCs, (n = 5, p < 0.05 one way ANOVA with post-hoc Tukey test). [file Image_4.TIF]
